# Analysis of Differences in Running Demands between Official Matches and Transition Games of Young Professional Soccer Players according to the Playing Position

**DOI:** 10.5114/jhk/175339

**Published:** 2024-02-17

**Authors:** Jose A. Asian-Clemente, Alberto Rabano-Muñoz, Luis Suarez-Arrones, Bernardo Requena

**Affiliations:** 1 Department of Sport sciences, Universidad Pablo de Olavide, Sevilla, España.; 2 Football Science Institute, Granada, España.; 3 FC Lugano, Performance Departament, Lugano, Switzerland.

**Keywords:** soccer, match demands, training load, high-speed running, locomotor activities

## Abstract

The aim of this study was to compare the running demands of transition games (TGs) and official matches, analysing their requirements according to the performance of each position. An observational design was used to examine the activity of 20 soccer players during official matches and TGs. GPS technology was used to monitor the total distance covered (DC), distance at speeds between 14–17.9 km•h^−1^, 18–21 km•h^−1^, and above 21 km•h^−1^, peak speed, accelerations and decelerations above 2.5 m•s^−2^, and Player Load for both activities. All players were assigned to groups: centre-backs (CBs), fullbacks (FBs), defensive midfielders (DMFs), offensive midfielders (OMFs), wide midfielders (WMFs) and strikers (Ss). TGs showed greater total DC, DC 14–17.9 km•h^−1^, DC 18–21 km•h^−1^, DC >21 km•h^−1^, accelerations and decelerations >2.5 m•s^−2^, and Player Load (p < 0.01). CBs, FBs and Ss showed more DC, DC 14–17.9 km•h^−1^, DC 18–21 km•h^−1^, DC >21 km•h^−1^, accelerations and decelerations >2.5 m•s^−2^ and Player Load in TGs (p < 0.01). In the midfielder positions, transition game players showed greater DC 18–21 km•h^−1^, DC >21 km•h^−1^, accelerations and decelerations >2.5 m•s^−2^ than in matches (p < 0.05). DMFs showed higher total DC (p < 0.05) and WMFs greater DC and DC 14–17.9 km•h^−1^ (p < 0.01) in these drills. During transition games CBs showed greater DC 14–17.9 km•h^−1^ than FBs, and greater DC than Ss (p < 0.05). FBs performed more decelerations >2.5 m•s^−2^ than DMFs and OMFs (p < 0.05). TGs produced a homogenized load in soccer players, independent of their position, which exceeded the external load of official matches.

## Introduction

Soccer is an acyclic sport in which players perform intermittent activities characterized by the alternation of high and low intensity periods (Hader et al., 2019). Currently, a professional male soccer player covers on average 9–12 km per match, with large differences according to the positional role (Mohr et al., 2003, 2005). Some authors have demonstrated that a player performs between 150 and 250 brief, intense actions during a game (Bangsbo et al., 2007). These high-intensity actions are very important in goal-scoring opportunities and they are considered crucial to injury prevention in soccer players since in most of the goals, there is at least one powerful action by the scoring or assisting player, and an appropriate high-intensity ability reduces the risk of injury (Faude et al., 2012; Freeman et al., 2019; Malone et al., 2018).

Coaches and practitioners should plan appropriate exposure to high-intensity actions during training sessions, with the aim of either developing or maintaining players’ ability to perform intermittent effort as often as required during competition (Mohr et al., 2005; Svensson and Drust, 2005). The most common methods of training documented in professional soccer include high-intensity running training, ball-drills in the form of modified-sided games, and field-based drills that replicate the actions of match play (Buchheit and Laursen, 2013; Iacono et al., 2019; Kyprianou et al., 2019). Although high-speed running is effective from a physiological perspective, it is considered less appropriate and motivating by players (Arslan et al., 2020). The existing literature has shown that modified-sided games have some characteristics required to stimulate this capacity while small-sided games do not. Medium- and small-sided games with changes of the zone, or medium and large sides, seem more appropriate for that purpose (Asian-Clemente et al., 2021; Clemente et al., 2019; Lacome et al., 2018; Martin-Garcia et al., 2019), because the greater available space or the necessity to move to other areas enables players to achieve higher velocities.

It is well-established that more significant tactical, physical, and physiological improvements occur when specific training exercises closely replicate the demands of formal matches (Katis and Kellis, 2009). To reproduce high-speed running seen in matches, it is essential to incorporate offensive and defensive transitions, passes that seek available spaces between goalkeepers and defenses, and one-on-one situations (Asian-Clemente et al., 2021; Di Salvo et al., 2007). The most common field-based drills used by soccer coaches to train these situations are transition games (TGs) (Asian-Clemente et al., 2022). The literature has recently focused on tasks in which players continuously have to complete counter-attack situations using fast attacking and defensive transitions (Asian-Clemente et al., 2022, 2023a, 2023b). A recent study has reported that TGs require more high-intensity effort than modified side games and lead to reduced variability in the player load (Asian-Clemente et al., 2022). Moreover, the training load of TGs is responsive to time and space manipulation (Asian-Clemente et al., 2023a, 2023b). A recent study has also demonstrated that soccer players have maximal requirements during transitions (Bortnik et al., 2022).

In spite of their popularity, training tasks in TGs have not been well studied and it is not known how their requirements and running demands relate to competition situations. For this reason, the aims of this study were to compare the running demands of TGs and official matches and to analyse their requirements according to the individual running performance of particular positions.

## Methods

### Participants

**Figure 1:**
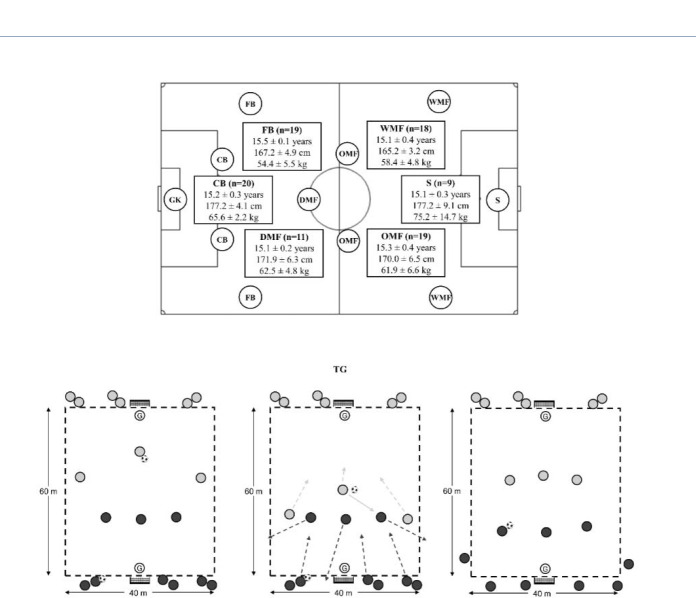
Sample and graphical representation. TG = transition games; CB = centre-back; FB = fullback; DMF = defensive midfielder; OMF = offensive midfielder; WM = wide midfielder; S = striker.

This study was performed with 20 field soccer players from a professional soccer team (goalkeepers were not included), who belonged to the professional academy of a Spanish team. Characteristics of players are shown in Figure 1. Their weekly routine consisted of one official match played during the weekend, five training sessions with duration of 80–120 minutes and one day off. Data were collected through daily workload monitoring during the team's training sessions, where player activities were measured. As a result, ethics committee clearance was not required; however, the experimental design adhered to the principles of the Declaration of Helsinki. Before players started the intervention, they attended a meeting that described the purpose of the study and the risks involved in the intervention and they provided signed consent to participate.

### Measures

External loads were measured using a GPS system (Catapult Vector S7, Catapult Sports, Australia) with a sampling rate of 10 Hz. In accordance with a previous study (Casamichana et al., 2012), the total distance (DC), distance covered between 14 and 17.9 km•h^−1^ (DC 14–17.9 km•h^−1^), distance covered between 18 and 21 km•h^-1^ (DC 18–21 km•h^−1^), distance covered at above 21 km•h^−1^ (DC >21 km•h^−1^) and peak speed were analysed. Other measures included the number of accelerations and decelerations above 2.5 m•s^−2^ (Acc > 2.5 m•s^−2^ and Dec > 2.5 m•s^−2^) and player load (Buchheit et al., 2014; Gomez-Carmona et al., 2019; Suarez-Arrones et al., 2016). Due to the difference in the duration of official matches and TGs, to compare the external load all variables were relativized per minute of play, except for peak speed, which was given as the highest value recorded.

### Design and Procedures

An observational design was used to examine the running activity of soccer players during competitive matches and TGs, using GPS technology. Data were obtained in the second half of the 2020−2021 season during the months from January to May.

Twelve official matches were analysed, including 3–12 measurements for each player (n = 96 match files). All matches were performed on outdoor artificial fields (98.8 ± 1.7 x 58.6 ± 1.9 m) using 11 players per side with a 1-4-3-3 formation (Figure 1). To compare the external loads between particular positions, all players were assigned to groups: centre-backs (CBs), fullbacks (FBs), defensive midfielders (DMFs), offensive midfielders (OMFs), wide midfielders (WMFs) and strikers (Ss). Analyses of each position are presented in Figure 1. Playing time was 2 x 45 min and only the time-motion data for players who participated in the entire game were retained for subsequent analysis.

TGs were evaluated on Wednesday (the day following the players’ day off). This day was chosen to avoid the influence of fatigue and high levels of loads were expected by the coaching staff. The sessions started with a 20-min warm-up, which included mobility, dynamic stretching and a soccer specific drill with passes and movements. The sessions were all conducted on the same training pitch and at the same time of day (16:00–18:00). The analysed drill is shown in Figure 1 and represents a transition game in which players had to attack and defend a specific counter-attack. During this game, players competed in groups of three players attempting to score/avoid a goal scoring. The task started with a group of three players facing each other. After the first move, regardless of whether it was a goal or a ball loss, three new players participated in the offensive role while the three defensive players rested and the three players who previously performed the attack had to defend, creating a new transition opportunity. This behaviour was repeated, involving all the players over 3 sets of 4 min (total time = 12 minutes). After each bout of 4 minutes, there was a period of 2 minutes of passive recovery. To ensure that all players had the same duration of activity, if the time elapsed and some players had not completed the same number of repetitions as their teammates, the drill continued until all participants had completed the same number of repetitions. All the drills were performed using the same pitch dimensions of 60 x 40 m. Additionally, players that suffered any injury during the intervention duration were excluded from the analysis.

To maximize playing time and guarantee maximal effort, soccer coaches supported the drill by introducing balls as needed and providing verbal encouragement. This TG was selected because it was commonly practiced by all the academy’s teams during their training programme, which reduced difficulty, uncertainty and complexity for players. Data obtained during TGs were also categorized in positional groups according to the individual roles adopted by players in the matches.

### Statistical Analysis

Data are presented as mean ± standard deviation (SD). All statistical analyses were performed using SPSS (version 19, SPSS Inc., Chicago, IL, USA). Data normality was verified using a Shapiro-Wilk test. The running activity of soccer players in official matches and training games was analyzed using a Student's *t*-test, and all data followed a normal distribution. A one-way analysis of variance (ANOVA) was used to compare differences in running activity during TGs between specific positions. Post-hoc tests were calculated using Bonferroni correction for multiple comparisons to identify significant differences. The level of statistical significance was set at *p ≤* 0.05. The standardized differences of effect size (ES, 95% confidence interval [95%CI]) in the selected variables were calculated. Threshold values for assessing magnitudes of the ES (changes as a fraction or multiple of baseline standard deviation) were > 0.20, 0.20, 0.60, 1.2 and 2.0 for trivial, small, moderate, large and very large effects, respectively (Hopkins et al., 2009).

## Results

Running activity in official matches and TGs is shown in Table 1. TGs showed significantly greater DC, DC 14–17.9 km•h^−1^, DC 18–21 km•h^−1^, DC > 21 km•h^−1^, the number of Acc > 2.5 m•s^−2^, the number of Dec > 2.5 m•s^−2^ and Player Load.

**Table 1:** Running demands of official matches and transition games.

	Matches	Transition games	ES	*p* value
DC (m•m^−1^)	107.1 ± 9.9	118.9 ± 8.4	1.52	<0.001
DC 14–17.9 (km•h^−1^)	13.5 ± 3.3	19.2 ± 2.3	1.72	<0.001
DC 18–21 (km•h^−1^)	4.7 ± 1.6	16.8 ± 3.4	3.41	<0.001
DC > 21 (km•h^−1^)	3.9 ± 2.1	21.3 ± 8.1	2.67	<0.001
Peak speed (km•h^−1^)	27.3 ± 2.0	27.0 ± 1.7	−0.05	0.63
Acc > 2.5 (m•s^−2^)	0.6 ± 0.2	1.7 ± 0.3	3.12	<0.001
Dec > 2.5 (m•s^−2^)	0.6 ± 0.2	1.2 ± 0.2	2.08	<0.001
Player Load (AU)	11.1 ± 1.7	13.0 ± 1.6	1.26	<0.001

Data are presented as mean ± SD. TG = transition games; ES = effect size; DC = total distance covered; Acc = number of accelerations; Dec = number of decelerations; AU = arbitrary units. All variables were relativized per minute of game except for peak speed, which is represented as the highest value

**Figure 2:**
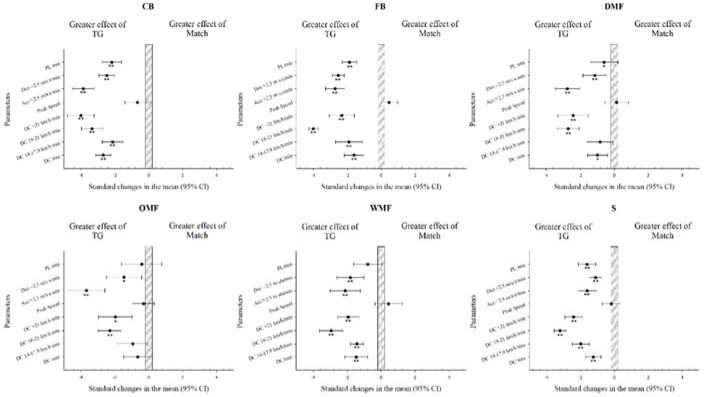
Comparison of the running demands according to individual positions in matches and transition games. TG = transition games; ES = effect size; CB = centre-back; FB = fullback; DMF = defensive midfielder; OMF = offensive midfielder; WM = wide midfielder; S = striker; DC = total distance covered; Acc = number of accelerations; Dec = number of decelerations; AU = arbitrary units; IC = confidence interval. * *p* < 0.05; ** *p* < 0.01. Data are presented as mean ± SD. All variables were relativized per minute of game except for peak speed, which is represented as the highest value.

Differences in the external load of official matches and TGs, according to individual positions, are presented in Table 2 and Figure 2. CBs, FBs and Ss achieved significantly higher levels of DC, DC 14–17.9 km•h^−1^, DC 18–21 km•h^−1^, DC > 21 km•h^−1^, #Acc > 2.5 m•s^−2^, #Dec > 2.5 m•s^−2^ and player load during TGs than in official matches. The three midfield positions (DMF, OMF and WMF) presented significantly greater DC 18–21 km•h^−1^, DC > 21 km•h^−1^, #Acc > 2.5 m•s^−2^ and #Dec > 2.5 m•s^−2^ values during TGs than in official matches. DMFs exhibited significantly more DC and WMFs showed more DC and DC 14–7.9km•h^−1^ during TGs in comparison with official matches.

**Table 2:** Running demands of official matches and transition games according to the particular positions.

		DC (m•m^−1^)	DC 14–17.9 (km•h^−1^)	DC 18–21 (km•h^−1^)	DC > 21 (km•h^−1^)	Peak speed (km•h^−1^)	Acc > 2.5 (m•s^−2^)	Dec > 2.5 (m•s^−2^)	Player Load (AU)
CBs	M	102.6 ± 8.5	12.4 ± 2.5	3.7 ± 1.4	2.7 ± 1.2	27.0 ±2.3	0.5 ± 1.4	0.5 ± 0.2	10.2 ± 1.2
	TG	126.1 ± 2.8	20.4 ± 1.9	18.6 ±3.2	26.3 ± 6.9	27.5 ± 0.6	1.7 ± 0.2	1.2 ± 0.2	13.4 ± 1.1
FBs	M	104.0 ± 8.6	13.1 ± 2.8	5.2 ± 1.5	5.2 ± 2.2	28.0 ± 1.5	0.6 ± 0.2	0.6 ± 0.1	10.9 ± 1.1
	TG	115.2 ±10.6	18.1 ± 0.7	16.7 ± 2.3	19.5 ± 9.4	26.4 ± 1.6	1.7 ± 0.4	1.4 ± 0.3	13.2 ± 1.1
DMFs	M	109.7 ± 8.3	13.3 ± 3.1	3.4 ± 1.1	2.1 ± 1.1	26.5 ± 2.2	0.5 ± 0.1	0.6 ± 0.1	10.5 ± 0.9
	TG	121.0 ± 6.8	18.4 ± 4.3	16.3 ± 3.3	20.7 ± 4.1	26.6 ± 1.1	1.7 ± 0.3	1.0 ± 0.4	12.2 ± 2.3
OMFs	M	116.7 ± 8.3	16.8 ± 3.3	5.8 ± 1.6	3.3 ± 1.6	26.3 ± 1.9	0.6 ± 0.2	0.6 ± 0.2	12.6 ± 2.0
	TG	118.6 ± 8.8	18.8 ± 4.4	14.7 ± 4.3	19.1 ± 10.0	27.4 ± 2.7	1.7 ± 0.1	1.1 ± 0.3	12.6 ± 0.9
WMFs	M	110.5 ± 6.4	13.6 ± 3.0	5.4 ± 1.3	5.3 ± 2.1	28.2 ± 1.6	0.7 ± 0.2	0.6 ± 0.2	12.1 ± 1.1
	TG	119.5 ± 6.5	18.4 ± 2.5	16.1 ± 4.4	21.7 ± 8.1	27.5 ± 1.9	1.6 ± 0.3	1.2 ± 0.2	13.8 ± 2.3
Ss	M	96.3 ± 7.2	10.4 ± 1.9	4.3 ± 1.0	4.0 ± 1.1	27.3 ± 1.5	0.7 ± 0.1	0.6 ± 0.1	8.9 ± 0.9
	TG	114.3 ± 9.2	20.1 ± 1.8	16.9 ± 2.7	21.5 ± 8.2	27.2 ± 1.8	1.6 ± 0.4	1.2 ± 0.4	12.5 ± 1.8

Data are presented as mean ± SD. M = Matches; TG = transition games; CB = centre-backs; FB = fullbacks; DMF = defensive midfielders; OMF = offensive midfielders; WM = wide midfielders; S = strikers; DC = total distance covered; Acc = number of accelerations; Dec = number of decelerations; AU = arbitrary units. All variables were relativized per minute of game except for peak speed, which is represented as the highest value.

**Figure 3:**
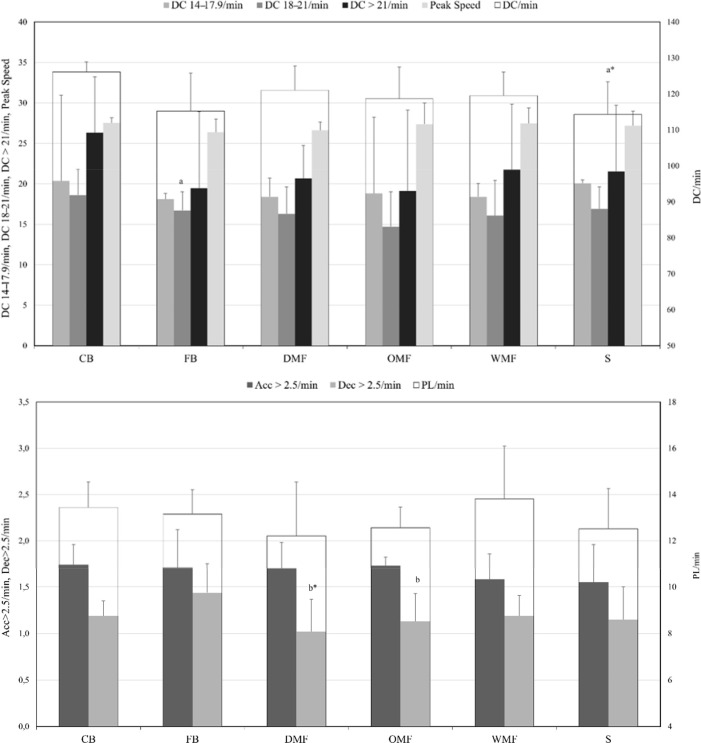
Comparison of the running demands of TG according to the individual positions. TG = transition games; CB = centre-back; FB = fullback; DMF = defensive midfielder; OMF = offensive midfielder; WM = wide midfielder; S = striker; DC = total distance covered; Acc = number of accelerations; Dec = number of decelerations; AU = arbitrary units; IC = confidence interval. a = statistically lower than centre-backs (*p* < 0.05), b = statistically lower than fullbacks (*p* < 0.05), * = moderate effect size. Data are presented as mean ± SD. All variables were relativized per minute of game except for peak speed, which is represented as the highest value.

Differences in running activity between specific positions during TGs are shown in Table 2 and Figure 3. CBs achieved significantly greater DC 14–17.9 km•h^−1^ than FBs, and greater DC in comparison with Ss. FBs presented significantly greater #Dec > 2.5 m•s^−2^ values than DMFs and OMFs.

## Discussion

The aims of this study were to compare running activity in young elite soccer players during TGs and official matches, and to describe movement patterns according to specific playing positions. The main findings show that TGs are suitable soccer specific drills to increase running activity of soccer players during official matches, achieving a similar load for all positions.

Comparing the demands of competitive matches to those of various training drills is an essential aspect of soccer training (Clemente et al., 2019). These comparisons enable coaches to replicate or even surpass actual match conditions (Owen et al., 2014). Nevertheless, there has been limited research on the comparison between matches and training (Gomez-Carmona et al., 2019). This was the first investigation to compare TGs with official matches in soccer players, showing that TGs achieved higher running demands above 14 km•h^−1^, acceleration-deceleration demands > 2.5 m•s^−2^ and player load in comparison with match-play activity. Despite the increased requirements of TGs, the peak speeds achieved in official matches and this training drill were similar.

The outcomes of this study are in line with previous investigations, which have affirmed that in small-, medium- and large-sided games as training tasks, players cover greater distances and achieve a higher number of accelerations and decelerations than in official matches (Asian-Clemente et al., 2022; Clemente et al., 2019; Gimenez et al., 2018; Owen et al., 2014). Our data contrast with these studies in relation to high-intensity running. While our data indicate that TGs are a suitable tool to overstimulate the higher-speed running requirements of official matches, modified sided games produce lower levels of high-speed running distance in comparison with official matches (Asian-Clemente et al., 2022; Clemente et al., 2019; Gimenez et al., 2018; Owen et al., 2014). Even the use of large-sided games (area relative per player = 300 m^2^ similar to the official matches) is insufficient to replicate match demands above 21 and 25 km•h^−1^ (Asian-Clemente et al., 2022). Previously, it has been established that the external load of TGs is determined by two main factors, i.e. the relative area per player and the length:width ratio (Asian-Clemente et al., 2023a). The studied TG was developed with a higher area per player (400 m^2^ vs. ≈300 m^2^), which could have led to greater demands for high-speed running. This behaviour has been demonstrated in modified sided games (Bujalance-Moreno et al., 2019). Official matches are played with a greater length:width ratio than the TG (1.7 vs. 1.5) and this may increase the speed of players (Asian-Clemente et al., 2023b). The structure of TGs, in which players in possession of the ball encounter fewer opponents and have more space in front of the opponent's goal, may have led to increased running demands.

Another remarkable aspect of this study is that official matches and TGs showed similar peak speeds. A previous study found that soccer players achieved higher peak speeds during official matches than during friendly matches, training matches and modified side games with a relative area per player of 300 m^2^ (Asian-Clemente et al., 2022). The discrepancies between these studies could be due to the samples used, because although both were carried out with young professional soccer players, the averages ages were different (15 vs. 20 years). Similarly, the dynamics of transition games, which involved continuous counter-attack actions, may have led soccer players to achieve their maximum peak speed. Previous research has suggested that larger transition games are associated with greater sprinting demands. Therefore, although this aspect requires further investigation, it appears that a pitch length of 60 m could be sufficient for training peak speed in soccer.

In an attempt to compensate for the lack of high-speed running in the commonly used modified sided games, alternative drills are being investigated in the literature. For example, a recent study showed that small-sided games in which players had to change to another zone during the exercise resulted in more DC > 18–21 km•h^−1^, DC > 21 km•h^−1^, and greater numbers of accelerations and decelerations (Asian-Clemente et al., 2021). TGs may also provide useful alternatives since a recent study demonstrated that TGs demanded more DC 18–21 km•h^−1^, DC > 21 km•h^−1^, and produced higher peak speeds and Acc > 2.5 m•s^−2^ than large-sided games and small-sided games where players had to change their playing space during the game (Asian-Clemente et al., 2022). As a result of the existing knowledge about TGs and the new information reported in this study, performing repeated transition sequences with a reduced number of players (3 vs. 3) in a large space (60 x 40 m) appears to be a useful way to exceed the requirements of official matches in a specific counter-attack context.

When individual positional responses were analyzed, the results showed similarities to those obtained without distinguishing between positions, indicating greater external loads in TGs than in competition for all positions, with the exception of peak speed, which remained the same. Previous studies also found differences in the positional running demands of training tasks and matches (Dellal et al., 2012; Martin-Garcia et al., 2019, 2020). Although those studies affirmed that the external load could over- or under-stimulate players in each specific position depending on the soccer drill (i.e., more high-speed running during larger-sided games or more accelerations/decelerations during smaller-sided games for all positions) (Martin-Garcia et al., 2019, 2020), TGs demonstrated over-stimulation of the most demanding variables of external loads for all positions. Soccer coaches could use these drills in mid-week sessions or during more intense soccer sessions when they look for high specificity that encourages learning transference of technical-tactical principles (Delgado-Bordonau and Mendez-Villanueva, 2012) under a demanding training load. Although positional roles have an influence on running performance during official matches (Di Salvo et al., 2007, 2009), TGs did not show differences between specific playing positions in terms of distance covered at different speeds. Considering acceleration and deceleration demands, the results are in line with previous studies, showing that FBs execute more decelerations than OMFs and DMFs (Dalen et al., 2016, 2019), but with no other differences between particular playing positions. The lower positional requirements of TGs could ensure greater equality between the external load on players during the exercises, independently of their positions. This, together with the need to use training to replicate the structure of competition and to overload non-starter players within a soccer-specific context, especially in high-speed running (Akenhead et al., 2016; Martin-Garcia et al., 2019; Stevens et al., 2017), suggests the TGs may be a suitable exercise to achieve these aims.

This study has some limitations that should be considered before applying the results. For example, the studied TGs, although they have the same playing area than matches, involve a reduced number of players. Therefore, it could be interesting to conduct future studies that would explore TGs with varying numbers of players and of different pitch sizes. Also, the internal response of soccer players was not monitored, and futures research could analyze this during TGs. Similarly, physical variables were monitored independently of technical and tactical aspects, thus future works should analyze these components in TGs.

## Conclusions

This study provides useful and novel findings for coaching staff about the use of TGs and their running requirements in comparison to match demands. It has been demonstrated that TGs have higher demands in terms of distance covered, DC > 14 km•h^-1^, neuromuscular load, and Player Load compared to official matches. Similarly, regardless of their playing position, soccer players achieve higher speeds and experience greater accelerations-decelerations loads during TGs. Finally, it can be affirmed that TGs are suitable soccer specific drills to achieve a uniform workload among soccer players, regardless of their positions.
